# From Athletes to Astrophysicists: Gender Differences in Patterns and Predictors of Career Aspirations in Pre-Adolescence

**DOI:** 10.3390/socsci5010005

**Published:** 2016-01-28

**Authors:** Brea L. Perry, Edward W. Morris, Tanja C. Link, Carl Leukefeld

**Affiliations:** 1 Department of Sociology, Indiana University, Ballantine Hall 744, 1020 East Kirkwood Ave., Bloomington, IN 47405, USA; 2 Department of Sociology, University of Kentucky, 1569 Patterson Office Tower, Lexington, KY 40506, USA; 3 Department of Sociology & Criminal Justice, Kennesaw State University, 402 Bartow Ave, Kennesaw, GA 30144, USA; 4 Department of Behavioral Science, University of Kentucky, 111 Medical Behavioral Science Building, Lexington, KY 40506, USA

**Keywords:** career aspirations, gender differences, masculinity, intersectionality, middle school

## Abstract

This paper adds to research on girls’ growing educational advantage by examining gender differences in career paths. Using baseline data from an intervention study (TRY-IT!) targeting 265 sixth-graders in Title I schools, our research traces adolescent career aspirations by gender, race and class. Additionally, we investigate whether girls and boys exhibit differential sensitivity to environmental risk and protective factors that shape career and educational aspirations. We find that the career choices of boys vary more widely by social context, including socioeconomic status, race, and academic resources. Specifically, among youth with fewer social and academic advantages, girls aspire to more practical careers and careers which require higher levels of educational attainment relative to boys. The findings reveal how sources of inequality such as race and class shape gendered aspirations and complicate gender inequality. We reason that boys’ choices are more volatile and socially contingent because of the emphasis on high-status careers as a signifier of masculinity.

## 1. Introduction

Throughout Western societies, girls are surpassing boys in most areas of educational achievement and attainment [1,2]. This “new” gender gap has prompted the reassessment of traditional concepts of gender inequality. Nevertheless, socially dominant gender construction still determines girls to be subordinate to their male peers (see, for example, 2000). Because women and girls represent a subordinate gender group, it appears contradictory that they would hold clear advantages within a vast social institution such as education. Using baseline data from an intervention study (TRY-IT!) targeting 265 sixth-graders in schools with a high proportion of students qualifying for free or reduced-price lunches (*i.e.*, Title I schools), this paper adds to research on girls’ growing educational advantage by considering what this trend means for career paths, thereby addressing a gap in our current knowledge about early gender differences in career and educational aspirations While girls are now more likely to matriculate in higher education [3] we know less about what types of career and educational trajectories girls and boys envision in adolescence.

Our research traces adolescent career aspirations by gender, race and class and classifies those careers according to their attainability (*i.e.*, number of available positions in that field) and educational requirements. Even though prior research has documented that career aspirations are seldom realized [4], others have pointed out that individuals with high aspirations are indeed more likely to end up in high status occupational fields [5,6]. Furthermore, existing literature has confirmed the significance of SES (Socioeconomic status) on occupational aspirations [5,7,8]. We find that among boys and girls with fewer social and academic resources, girls aspire to more practical careers and careers which require higher levels of educational attainment. The findings reveal how sources of inequality such as race and class shape gendered aspirations and complicate gender inequality.

## 2. Background

One of the key findings of the gender gap in education is that it varies significantly by race and class status [9,10]. Among more advantaged students, gender gaps in attainment and achievement are slight, or insignificant. However, these gaps grow in magnitude among groups disadvantaged by race, class, and school composition [11,12]. Ethnographic evidence suggests that poor and working class boys deem academically oriented behavior as un-masculine, and thereby avoid putting effort into their schoolwork [13,14]. Similarly, research finds that Black urban boys interpret school-focused identities as “soft” and insufficiently streetwise [15,16]. Partially because of this ambivalence toward schooling, lower income boys often aspire to manual labor or athletic careers [17].

Recent explanations of these gender differences by race and class claim that boys might be more vulnerable than girls to social influences. Research shows that peer groups strongly influence boys’ educational attitudes. Legewie and DiPrete [12] find that boys’ academic behavior is more affected than girls’ academic behavior by the socioeconomic context of the school environment. In resource-rich educational settings, which foster a strong orientation towards learning, boys and girls show similar academic outcomes. However, in resource-depleted settings, which do not have a strong learning focus, boys’ outcomes decline relative to girls. The authors reason that girls’ peer groups are more resilient to lack of resources because they do not value resistance to school as a gender signal. Boys are more likely to view institutional defiance as a demonstration of masculine power in settings where they perceive to be disempowered in other ways [17]. Because girls are not socialized to predicate their gender on efforts to “exert control and resist being controlled” ([18], p. 61), their peer groups do not emphasize demonstrations of power. This frees disadvantaged girls to exhibit more academic interest and effort.

Such peer group influences also emerge through race. Anderson’s research [19] for example, shows how young Black men are pushed, both through stereotypes and through perceived rewards, to exhibit a tough, streetwise exterior. This stance conflicts with academic institutions, and can manifest in defying the authority of these institutions [20]. In some cases, Black boys may find it difficult to escape stereotypes of Black masculinity that can put them at odds with school. In a study of a school integration program, Ispa-Landa [21] finds that Black boys bussed to suburban schools were embraced by White peers who saw them as cool. However, the coolness of these boys hinged on peer expectations to demonstrate a tough, street-smart façade, which conflicted with academic demands. Some boys even explained that they did not see themselves as tough, cool, and intimidating, but they nevertheless affected these cues because of the social cachet they garnered.

Wilkins [22] finds that similar expectations of Black men follow them when they attend college. In her study of college adjustment among Black and White first-generation college students, Black men expressed ambivalence about the cultivation of cool identities in college, a site where the “costs of coolness increased” compared to high school ([22], p. 184). For example, several young Black men in the sample complained that White peers assumed they were there on athletic scholarships or thought they were more interested in athletics than academics. This made it difficult for these men to cultivate academic identities because stereotypes of Black masculinity constantly pushed them into a box defined by perceived athletic ability.

One shortcoming of research on the gender gap is that it tends to focus on why boys are falling behind instead of asking why girls are doing so well [23]. These findings underscore the resilience of disadvantaged girls at the same time that they expose the pitfalls facing disadvantaged boys. But the educational perceptions of poor and minority girls also add important dimensions to our knowledge of how race and class interact with gender. Studies find that African American girls tend to be less fettered by low self-esteem and self-efficacy than White girls [24,25]. Interestingly, although low-income White girls might suffer from low self-esteem and self-efficacy, this does not appear to hinder their academic progress. Morris [17] who explored the gender gap in educational achievement at two low-income high schools—one rural and predominantly white, the other urban and mostly African American found that the self-deprecating attitudes of lower income rural girls actually catalyzed academic efforts that increased their achievement compared to their male peers. These girls appeared to adopt a “growth-based” mindset that their achievement depended more on effort than intrinsic talents [26].

Drawing from this previous research, we suggest that gender differences in career choices are influenced by boys’ efforts to claim maximum status dividends based on perceived resources available. We predict that boys’ choices will hinge more strongly on their social environments, revealing how race and class shape career choices through gender. Schrock and Schwalbe [27] argue that masculinity is the method through which males signify a “masculine self” which claims power within the gender order. Morris [17] extends this view to understand how disadvantaged boys navigate various perceived challenges and resources for attaining gender power. Race and class interact with gender to produce different challenges and resources, while the ultimate goal of masculine status remains.

This framework provides an explanation for why boys’ career choices tend to be more extreme than girls’ career choices. Boys learn that the “breadwinner” is a major feature of masculinity, and they anticipate careers to attain this status based on the resources they believe they possess. Low-income African American boys might view athletics as the most viable (in their minds) path to this goal, while affluent boys might favor careers in math and science. Importantly, this framework also allows for a clearer window into girls’ choices. Girls will be less flamboyant in career aspirations because gender for them is not as highly centered on career status. Moreover, in disadvantaged communities, girls realize that economic reliance on men is unlikely [28]. While this issue is not limited solely to low-income communities, it is arguably a larger issue there due in part to the well-documented effects of decades of mass incarceration which has affected disadvantaged neighborhoods disproportionately and created a void of marriage-eligible males in the community who would be in an economically attractive position to be the main breadwinner of a family [29]. This may reinforce the resolve of disadvantaged girls’ to pursue careers that require academic attainment. Thus, compared to disadvantaged boys, disadvantaged girls should make more practical career choices and should show more resilience to risk factors.

Despite growing interest in the gender gap in education, little research has examined girls’ relative advantage through an intersectionality lens. This trend necessitates a reassessment of traditional perspectives on gender, as well as an examination of how gender operates differentially in the context of other forms of disadvantage [30,31]. To understand what is driving the gender gap in education, it is critical to determine which groups of boys are at risk and why. Girls’ aggregate educational advantages may be attributable to a qualitatively unique set of experiences and constraints faced by boys of color and those in low socioeconomic status groups, in particular, combined with relative gains made by their similarly-disadvantaged female peers. Yet, this perspective is largely absent in existing educational research [25].

The goal of the current study is to examine the gender gap in education through an intersectional framework, investigating the consequences of multiple marginalized identities for early career aspirations and their potential implications for academic trajectories. Additionally, we investigate whether girls and boys exhibit differential sensitivity to environmental risks and protective factors that shape career and educational aspirations, highlighting the resilience of disadvantaged girls. This research is guided by the following questions:

1. What career fields do sixth grade students in low-income schools aspire to enter as adults?
2. Are there gender differences in reported career aspirations and educational requirements for aspired careers?
3. Are there gender differences in the effects of race, poverty status, and academic resources (*i.e.*, self-esteem and self-concept related to academic performance, and opportunities for enrichment outside of school) on career aspirations?

## 3. Methods

### 3.1. Sample

Analyses for the current paper utilize baseline data for four cohorts of TRY-IT! participants and controls (*n* = 291). TRY-IT! was an intervention study focusing on helping students use technology to improve their understanding of biomedical science. Middle school students in the treatment group participated in intensive, two-week summer camp programming and monthly science workshops during the academic year over a period of three consecutive years. See [25,32] for a detailed description of the intervention and its efficacy. The baseline data analyzed in the current study, however, was collected prior to any intervention or randomization into control *versus* treatment groups.

Recruitment targeted schools serving large numbers of children from low-income and minority backgrounds in Kentucky. All sixth graders from four schools with a high proportion of students qualifying for free or reduced-price lunches (*i.e.*, Title I schools) were sent applications inviting participation. Consistent with federal IRB requirements, informed consent was sought from all participating students. Data were collected from every consenting student who applied for the program beginning in 2007. The current analysis used data on all applicants (prior to any intervention) that completed the career aspirations section of the paper-based survey (*n* = 265; 91% of the original sample). About 57% of survey respondents were girls and 43% were boys. The sample ranged in age from 11 to 13, with most being 11 (52%) or 12 (45%) years old at baseline. Racial and ethnic minorities were over-represented, with 39% being White, 34% Black, and 26% being some other race or multiracial. About 44% of the sample reported a household income of less than $30K, and about 31% reported incomes at or above $60K. The racial and socioeconomic characteristics of the sample reflected the sampling frame targeting low-income schools.

### 3.2. Measures

#### Career aspirations

Career aspirations were measured using an open-ended item reading: “In the space below, please describe your thoughts about your future career and/or a career that appeals to you (in any field)”. These open-ended responses were coded and organized by field (See Table 1). Since about 40% of students named more than one career, these were analyzed as a series of potentially overlapping binary dependent variables. Each career was also coded for educational requirements (in years), as indicated by the Bureau of Labor Statistics. Where students named multiple careers, the average required years of schooling was calculated.

#### Socio-demographic characteristics

Gender was measured using a dichotomous variable (1 = female). Race was measured using a series of dichotomous variables created from an item asking respondents to self-report which category best describes their race. Options were “White”, “African American”, “Hispanic/Latino”, “Native American”, “Asian”, and “Other”. The Native American and Other categories were too small to include as separate categories. In addition, because coefficients for Hispanic/Latino and Asian did not differ significantly from coefficients for White in regression models, Black students were compared to all other races and ethnicities. Parents’ educational attainment was measured in years using the mean of mother’s and father’s years of schooling. Where information on one parent was missing (usually father), the other parent’s educational attainment was used. Participation in the free or reduced-price lunch program was used to identify families in poverty and measured using a dichotomous variable (1 = yes)

#### Academic attitudes and resources

Three measures of academic attitudes were included in models to identify gender differences and to determine the extent to which these influence career aspirations. First, a 10-item subscale of the Hare Self-Esteem Scale (HSS) [33] measured self-worth or self-pride in the school domain. It included such items as “School is harder for me than most other people”. Each item was measured on a four-point scale. The scale mean was included in regression models, and ranges from 1–4 (higher values indicated more self-esteem). Second, confidence in college prep coursework was measure using a 10-item scale [34]. It asked participants to rate their confidence in their ability to successfully complete a list of nine advanced high school courses, including calculus, chemistry, geometry, and computer science. Each item was accompanied by a ten-point scale ranging from “completely unsure” to “completely sure”. The scale average with a potential range of 1–10 was used in analyses, with higher values signifying more confidence. Third, a 5-item subscale from the Attitude Toward Science Inventory [35] was employed to assess science self-concept (e.g., “Science is easy for me”). Responses were measured on a 5-point scale. The scale mean was used (potential range of 1–5), with higher values indicating a more positive self-concept. In addition, a count variable for academic resources was created indicating the number of enrichment opportunities students were exposed to in the home or outside of school (e.g., visiting a museum, participating in academic clubs, having a telescope in the home, subscribing to a newspaper, *etc*.). It ranged from 1–13.

### 3.3. Analytic Strategy

The principle goal of these analyses was to examine patterns and predictors of career aspirations among sixth graders in low-income schools. A secondary objective was to explore gender differences in the impact of risk factors and resources on career aspirations. Descriptive and bivariate statistics were used to identify gender differences across a variety of baseline indicators. A series of *t*-tests for continuous variables and chi-square tests for categorical variables were conducted to determine whether gender differences were statistically significant.

Logistic and OLS (Ordinary Least Squares) regression were used to examine the effects of independent variables on aspired career field (categories not mutually exclusive) and years of schooling required to obtain one’s career goals. Scales were x-standardized to facilitate interpretation. Main effects of socio-demographic characteristics were estimated with a series of seven regression models (one for each dependent variable). Academic attitudes and resources were then individually added to each model. Finally, interaction terms for gender × race, gender × SES, or gender × academic attitudes and resources were added one at a time to determine whether the effects of predictors vary by gender. Full regression results with interaction terms are available by request. Significant gender interactions were depicted using graphs of predicted probabilities or predicted values with bars representing confidence intervals. The Delta method [36] was used to assess the significance of interactions in logit models, while the *p*-value for the interaction term was used for OLS regression models.

## 4. Results

Students in the sample reported a wide range of career aspirations. The most common were health and medicine (32%) and science and technology (29%). Substantial proportions of students were also interested in careers in creative and performing arts (20%), law and criminal justice (17%), professional sports (11%), and K-12 education (8%). These careers were heterogeneous with respect to educational requirements, ranging from high school diploma or less (e.g., visual artist, professional athlete; 16%) to an advanced degree such as an MD or PhD (e.g., doctor, lawyer, veterinarian; 50%). Careers differed with respect to the feasibility of attainment, as indicated by the number of available jobs. Some careers were highly unattainable (e.g., fashion model, with 1240 available jobs in the U.S. in 2012), while others were more practical (e.g., K-12 teacher, with 3,089,900 jobs in 2012). In general, professional athletics and creative and performing arts were the career fields with the least opportunity.

### 4.1. Bivariate Gender Differences

Table 2 presents bivariate gender differences in socio-demographic characteristics, academic resources, and career aspirations. There were no significant gender differences in race/ethnicity or socioeconomic status in this sample, suggesting that these factors did not confound gender differences in key variables. Girls and boys were similar with regard to academic attitudes and resources, with one exception. Girls reported slightly higher school self-esteem than boys (3.35 *versus* 3.18; *t* = −2.61, *p* < 0.01).

The largest gender differences were reflected in the types of careers chosen by girls and boys, as well as the educational requirements for those careers. Girls were disproportionately likely to aspire to careers in health and medicine (*X*^2^ = 15.79, *p* < 0.001), and boys in science and technology (*X*^2^ = 31.03, *p* < 0.001). About 43% of girls named careers in health and medicine, compared to only 19% of boys. Conversely, 46% of boys expressed interest in careers in science and technology, but only 15% of girls did. Girls were also significantly more likely to aspire to careers in creative and performing arts (25% *versus* 13%; *X*^2^ = 5.85, *p* < 0.05) and teaching K-12 (13% *versus* 2%; *X*^2^ = 9.62, *p* < 0.01). Lastly, boys were disproportionately inclined to report interest in professional athletics (*X*^2^ = 15.63, *p* < 0.001), with 20% of boys choosing this career path compared to only 5% of girls.

Results also suggested important gender differences in the educational degree requirements for aspired careers. Focusing on the career with the highest degree requirements (when multiple careers were named), 58% of girls expressed interest in careers requiring an advanced degree (e.g., MA, PhD, JD) compared to only 39% of boys. However, boys were more likely than girls to choose careers requiring a 4-year Bachelor’s degree (38% *versus* 25%) or only a high school diploma (19% *versus* 13%). Interest in careers requiring an Associate’s degree was about equal across gender. These significant differences (*X*^2^ = 9.73, *p* < 0.05) indicated that while most of the students in the sample aspired to careers requiring a Bachelor’s degree, girls were disproportionately likely to express career interests that would lead them to obtain an advanced degree. Overall, the bivariate analyses demonstrated substantial early gender stratification in career aspirations that could affect educational trajectories.

### 4.2. Multivariate Regression Models

Results from multivariate regression models predicting aspired career field are located in Table 3. As anticipated based on bivariate results, girls had about three times greater odds of expressing interest in careers in health and medicine than boys (*p* < 0.001), and this was not explained by differences in academic attitudes and resources. Black students were less likely to choose these careers than students from other racial or ethnic groups (OR = 0.47, *p* < 0.05), and this finding also held after adding academic attitudes and resources to the model. For each standard deviation increase in school self-esteem, students were predicted to be 43% more likely to choose health and medicine careers (*p* < 0.05).

Focusing on science and technology careers, girls were estimated to be significantly less likely to choose these careers relative to boys (OR = 0.21, *p* < 0.001), and this result held in the full model. Students in the free and reduced lunch program were predicted to have lower odds of naming science and technology careers than their more advantaged peers (OR = 0.49, *p* < 0.05), but this effect was explained by SES differences in academic attitudes and resources. A standard deviation increase in positive science self-concept was associated with a predicted 116% increase in the odds of aspiring to science and technology careers (*p* < 0.001), and each additional enrichment opportunity outside of school was associate with a 29% increase (*p* < 0.001).

With respect to other careers, girls were predicted to be more likely than boys to aspire to careers in creative and performing arts (OR = 2.15, *p* < 0.05) and K-12 education (OR = 7.38, *p* < 0.001), but less likely to name careers in professional sports (OR = 0.16, *p* < 0.001). Black students were also estimated to be disproportionately likely to aspire to careers in professional sports (OR = 3.01, *p* < 0.01). Having more academic attitudes and resources to draw on seemed to channel students away from careers in the arts and athletics. A standard deviation increase in confidence in ability to complete college prep courses was associated with an estimated 31% lower odds of expressing interest in creative and performing arts careers (*p* < 0.05), while an increase in science self-concept was related to 45% lower odds of naming a career in professional sports (*p* < 0.05).

Findings pertaining to predictors of educational requirements for career aspirations are presented in Table 4. For these analyses, the dependent variable was required years of schooling. Being a girl was associated with a predicted 1.74 year increase in years of schooling if career aspirations were to be achieved (*p* < 0.001). Black students’ career aspirations were estimated to result in 1.30 fewer years of education compared to white students (*p* < 0.01), though this finding was largely explained by racial differences in academic aspirations. A standard deviation increase in confidence in ability to complete college prep courses was associated with an estimated additional 0.68 years of education to achieve career aspirations (*p* < 0.01).

### 4.3. Interactions: Gender Differences in Effects of Covariates

**Figure 1:**
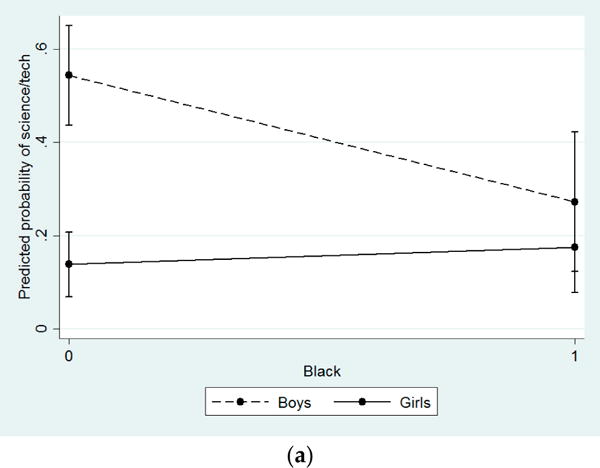

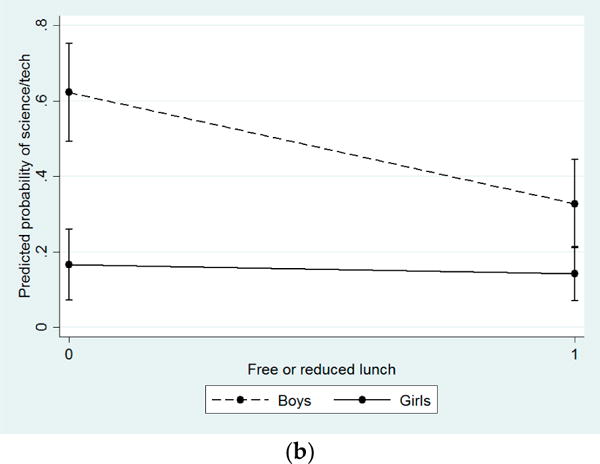
Predicted probability of aspiring to a career in science or technology by race, SES, and gender. (**a**) Effect of minority status (race) on to a career in science or technology among boys and girls; (**b**) Effect of receiving free or reduced lunch (SES) on aspiring to a career in science or technology among boys and girls. Note: Interactions significant at *p* < 0.05.

Interaction terms were added to baseline models to determine whether there were gender differences in the effects of socio-demographic and academic attitudes and resources on career aspirations. Only statistically significant interactions were depicted in graphs of predicted probabilities and predicted values. As shown in Figure 1, the adverse effects of minority status (OR = 0.36) and being on free or reduced lunch (OR = 0.31) on the odds of being interested in science and technology were only present for boys. Among students who could afford to pay for their lunch, boys’ predicted probability of being interested in science and technology careers was 0.55 compared to only 0.14 for girls. Likewise, among White students, the predicted probability of naming a science or technology career was 0.62 for boys and only 0.18 for girls. Thus, gender differences among Black students and those on free or reduced lunch were substantially reduced and non-significant.

**Figure 2:**
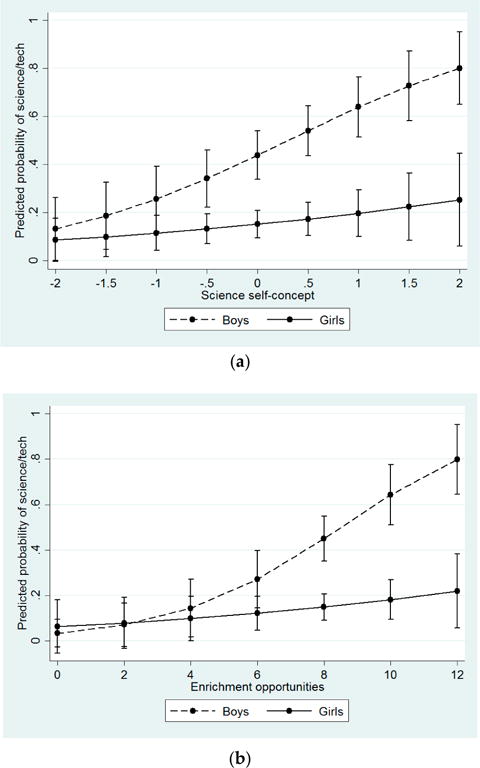
Predicted probability of aspiring to a career in science or technology by academic attitudes and resources and gender. (**a**) Effect of science self-concept (academic attitudes) aspiring to a career in science or technology among boys and girls; (**b**) Effect of enrichment opportunities (resources) on aspiring to a career in science or technology among boys and girls. Note: Interactions significant at *p* < 0.05.

Similar interactions were evident for the effects of academic attitudes and resources on interest in science and technology careers (See Figure 2). Specifically, increases in science self-concept (OR = 2.30), confidence in ability to complete college prep courses (OR = 1.41), and enrichment opportunities (b = 1.49) were associated with increases in the predicted probability of aspiring to careers in science and technology for boys, but not for girls. At and above the sample means for these resources, boys were significantly more likely than girls to express interest in science and technology careers, but there were no gender differences among students with lower levels of academic resources.

**Figure 3:**
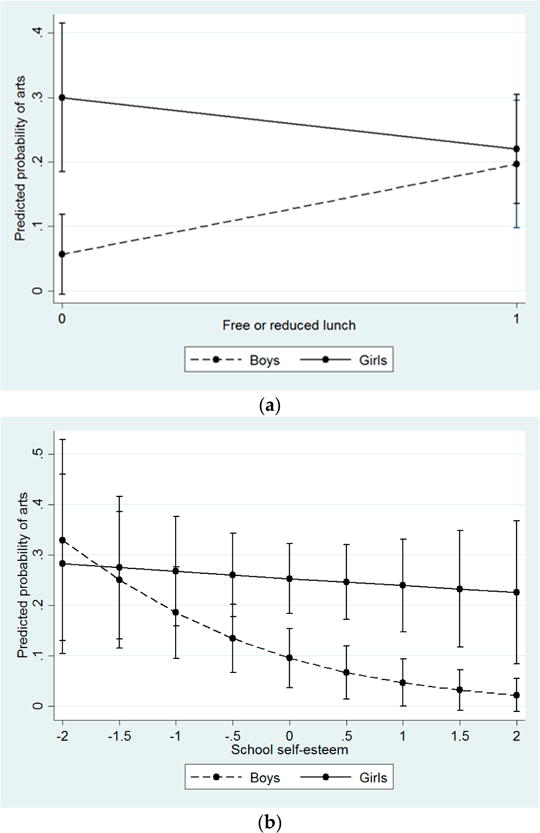

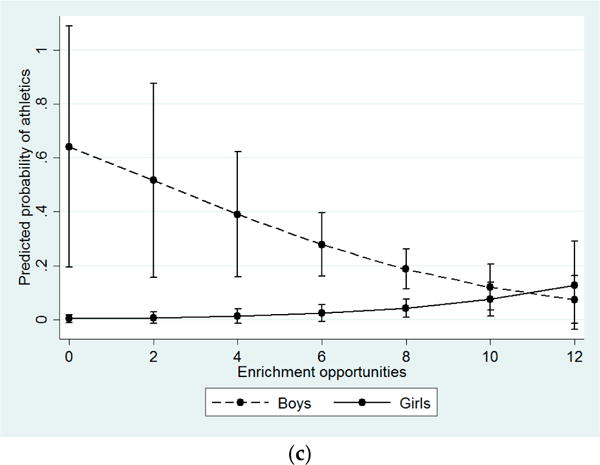
Predicted probability of aspiring to a career in arts or athletics by SES, academic attitudes and resources, and gender. (**a**) Effect of receiving free or reduced lunch (SES) on aspiring to a career in arts or athletics among boys and girls; (**b**) Effect of school self esteem (academic attitudes) on aspiring to a career in arts or athletics among boys and girls; (**c**) Effect of enrichment opportunities (resources) on aspiring to a career in arts or athletics among boys and girls. Note: Interactions significant at *p* < 0.05.

With respect to careers in fields with less opportunity, there were also significant gender interactions (See Figure 3). Among boys, being on free or reduced lunch was associated with an increase in the likelihood of aspiring to a career in creative and performing arts (OR = 3.40), but SES had an opposite and more modest effect for girls (OR = 0.51). For higher SES students, the predicted probability of choosing a career in creative and performing arts was 0.30 for girls and only 0.06 for boys, but this probability was nearly equal (around 0.20) among boys and girls on free or reduced lunch. Likewise, among sixth grade boys, additional academic attitudes and resources were associated with decreasing propensity to choose careers in the arts (*i.e*., school self-esteem; OR = 0.46) or professional athletics (*i.e*., enrichment opportunities; OR = 0.76), but this protective effect was diminished or absent for girls. Across these outcomes and predictors, gender differences were present at high levels of socioeconomic and academic resources, but not at lower levels.

**Figure 4:**
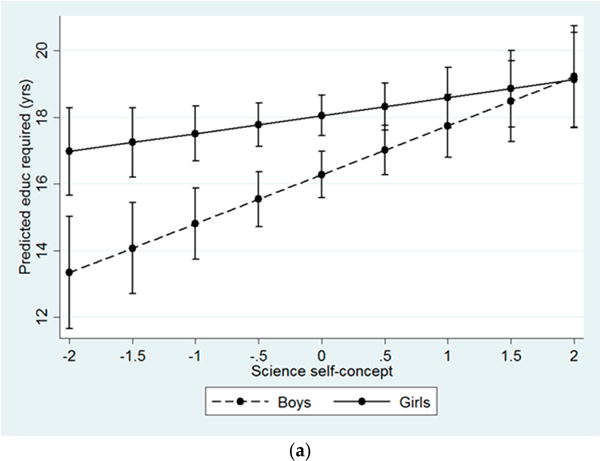

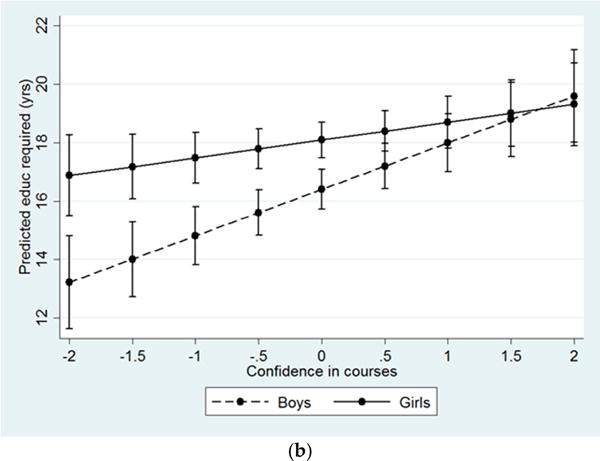
Predicted value of years of education required for career aspirations by academic attitudes and resources and gender. (**a**) Effect of science self-concept (academic attitudes) on years of education required for career aspirations among boys and girls; (**b**) Effect of confidence in ability to complete college courses (resources) on years of education required for career aspirations among boys and girls. Note: Interactions significant at *p* < 0.05.

Finally, Figure 4 depicts predicted values of years of education required for students’ career aspirations by gender and academic attitudes and resources. Among boys, the effects of each standard deviation increase in science self-concept (b = 1.46) and confidence in ability to complete college prep courses (b = 1.59) on career-dependent years of education are fairly large in magnitude. However, these effects are comparatively weaker and non-significant among girls (b = 0.52 and b = 0.86, respectively). At low levels of academic attitudes and resources, there are significant gender differences in years of schooling required for aspired careers, but these gender differences completely disappear at high levels of academic attitudes and resources.

In sum, the findings revealed several instances in which career aspirations varied between girls and boys depending on their race, low-income status, and self-reported academic attitudes and resources available to them.

## 5. Discussion

In sum, girls and boys in the TRY-IT! baseline sample expressed divergent career aspirations as early as sixth grade, and differences in educational requirements for gendered aspirations could potentially manifest in gender disparities in educational attainment. Academic attitudes and resources such as school self-esteem and enrichment opportunities also played an important role. Higher academic attitudes and resources were linked to career aspirations in health and medicine or science and technology, and reduced the odds of aspiring to less attainable (and more impractical) careers in creative and performing arts and professional athletics. Coupled with findings on gender differences in career fields of interest, academic attitudes and resources seemed to channel more confident and advantaged girls into health and medicine and boys into science and technology, and less resource-rich girls into creative and performing arts and boys into professional athletics.

However, the influence of gender became clearer in results from interaction models. In domains where boys were already advantaged (*i.e*., career aspirations in science and technology), additional resources gave them a substantial leg up over girls. That is, there were negligible gender differences among students with fewer resources but large differences favoring boys among those with more resources. In contrast, in cases where boys were disadvantaged relative to girls (*i.e*., educational requirements for career aspirations, careers in professional athletics), additional socio-demographic or academic resources evened the playing field for boys. Most importantly, *regardless of the outcome, boys’ aspirations were more strongly influenced by race, socioeconomic status, and academic resources than were girls’*. In other words, boys seemed to be more sensitive to their social and psychosocial environments than were girls.

### 5.1. Theoretical Implications

These findings highlight mechanisms through which racial and socioeconomic stratification is reproduced in the education system. Income and wealth, very much at the center of one’s social class, remain intrinsic to the educational opportunities one is afforded. As Fuller [37] discussed in her research, class imposes boundaries for all aspects of life, including ambitions and aspirations. In our research, youth in racial and ethnic minority groups and those from lower SES backgrounds reported career aspirations that differed systematically from those of their more advantaged peers. Their choices—“long shot” careers in professional athletics or performing arts—may reflect a perceived lack of opportunity to pursue more practical careers through conventional means (*i.e*., academic success and educational attainment). While young boys and girls from low-SES and racial/ethnic minority backgrounds typically have aspirations for wealth and prestige that mirror or exceed their more advantaged peers, they tend to have lower confidence in their academic abilities and likelihood of succeeding in educational pursuits [25]. Together, the combination of institutionalized goals and a lack of access to conventional pathways may lead young people to bet on non-academic talents, such as athletics, singing, or dancing ability, however impractical.

Consistent with other research on education [10,37,38], the findings on race and class differences in career aspirations were contingent on gender and emphasize the problematic nature of performances of masculinity for at-risk boys. Specifically, low-SES Black boys with fewer academic resources (e.g., lack of access to educational opportunities outside of school) were disproportionately likely to report career aspirations in athletics with low educational requirements and very little opportunity for achieving success. This finding is consistent with ethnographic research suggesting that poor and working class boys and Black urban boys perceive academically oriented behavior as un-masculine and inconsistent with a streetwise identity [13–16].

Moreover, relative to girls, boys were more strongly influenced by the socioeconomic and peer contexts of their academic environment, posing additional barriers to success among boys in resource-poor schools [12,17,18]. Disadvantaged boys, in particular, view resistance to school and success in athletics as strong signals of successful gender performance. At the same time, the “breadwinner” role and socioeconomic status attainment remain salient features of masculinity. Thus, it is unsurprising that boys with relatively little status and power would disproportionately emulate and seek to become professional basketball and football stars, who embody strength, masculinity, and often extreme upward mobility. Simultaneously, unlike more advantaged boys, they may not see opportunities for success in science or technology, or may not perceive these careers as sufficiently masculine. In short, in environments where rigid adherence to gender performance is particularly critical for the social status of men and boys, conventional pathways to occupational and socioeconomic achievement through higher education may be effectively cut off.

Another important insight emerging from these findings is the apparent resilience of young girls in the face of adversity. Overall, this sample of sixth grade girls in low-income schools fared better than their male peers with respect to school self-esteem, and aspired to careers with higher educational requirements. Moreover, lacking academic and socioeconomic resources did not influence the career aspirations of girls, but was devastating for boys. This pattern may relate to the performance of gender, which is less dependent on resistance to school for girls as it is for boys. In contrast, disadvantaged girls may see attainable middle class careers in health and medicine as a way out of their socioeconomic environment, particularly given the tenuous nature of relying on men for economic stability [28]. Existing research indicates that minority and low-SES girls possess high self-esteem and self-efficacy, or, alternatively, hold a growth-based mindset that relies on effort rather than intrinsic talents for success [17,24–26]. Consequently, they are less affected by under-resourced environments.

### 5.2. Limitations

The following limitations should be noted: First, this study was based upon a non-representative purposive sample. Although the opportunity to participate in the study and attend a free two-week summer camp focused on science and technology was presented to all students in selected schools, those who applied probably had a greater interest in science than those who did not. However, it is unlikely that the sampling strategy was related to the interactions identified since boys and girls had equal opportunity to participate. However, while differences in the effects of academic attitudes and resources across genders may not have been biased by the sampling method, predicted values were probably biased upward. In other words, estimates of interest in careers in health and medicine and science and technology, in particular, were probably greater than would be found in a probability sample. This suggests that these findings may have underestimated the gendered effects of academic attitudes and resources on career aspirations since this sample is probably skewed toward having more resources and interest.

Second, there is no way to know whether these early career aspirations actually shaped students’ educational choices in high school and beyond, or the degree to which these changed over time. Nevertheless, previous research [8,38] has documented that in some samples early aspirations have been significantly predictive of individual occupational trajectories. Thus, while it is impossible to know whether the career aspirations were consequential in our sample, there is evidence that it could be.

Finally, future analyses should investigate the importance and differences of self-reported second career choices as these might provide further insight to the occupational aspirations among different populations. That said, these data do provide an interesting snapshot of the early educational and career propensities of students from low-income schools who are at risk of underachieving. These findings demonstrated substantial stratification in the early career interests of girls and boys and, more importantly, gender differences in the extent to which psychosocial and economic resources could be used to improve one’s goals and prospects.

## 6. Conclusions

Broadly, results presented here suggest that career aspirations are stratified at the intersection of gender, race, and socioeconomic status. This study responds to calls for increasing attention to the complex effects of early life chances, revealing that the impact of race/ethnicity and socioeconomic disadvantage are contingent on gender [25]. We also contribute evidence to the growing body of literature documenting the troubled relationship between the American educational system and boys from low-SES and racial and ethnic minority groups [39,40]. In addition to consequences like poor academic performance and low educational attainment [3,12], resource-poor school environments may also lead boys to adopt career aspirations that ultimately reproduce disadvantage. Future research should explore psychosocial processes that cause boys to be more vulnerable to adverse environments while girls are comparatively more resilient.

## Figures and Tables

**Table 1:** Career aspirations of sixth graders in low-income schools (*n* = 265).

Career Field	Common Examples	% (n)
Health and medicine	Doctor, veterinarian, nurse	32.45% (86)
Science and technology	Biologist, engineer, computer programmer	28.68% (76)
Creative and performing arts	Singer, dancer, writer, visual artist	20.00% (53)
Law and criminal justice	Lawyer, judge, police officer, FBI agent	16.60% (44)
Professional athletics	Basketball player, football player	11.32% (30)
K-12 Education	Teacher (all specialties)	7.55% (20)

**Table 2:** Sample descriptive characteristics by gender, TRY-IT! (*n* = 265).

	Girls (*n* = 151)		Boys (*n* = 114)		

	Mean (SD)	% (n)	Mean (SD)	% (n)	*X*^2^ or *t*
*Race*					
White		39.07 (59)		39.47 (49)	3.19
Black		37.75 (57)		28.95 (33)	
Other or multiracial		23.18 (35)		31.58 (36)	
*Socioeconomic Status*					
Free or reduced lunch		60.26 (91)		53.51 (61)	1.21
Parents’ mean education (yrs)	13.45 (4.72)		13.85 (5.18)		0.64
*Academic attitudes and resources*^1^					
School self-esteem	3.35 (0.51)		3.18 (0.53)		−2.61 **
Science self-concept	3.82 (0.81)		3.99 (0.80)		1.60
Confidence in college prep courses	6.67 (2.37)		6.67 (2.32)		0.02
Enrichment opportunities	7.97 (2.04)		8.09 (2.21)		0.44
*Career aspirations* 2					
Health and medicine		42.38 (64)		19.30 (22)	15.79***
Science and technology		15.23 (23)		46.49 (53)	31.03***
Creative and performing arts		25.17 (38)		13.16 (15)	5.85*
Law and criminal justice		15.23 (23)		18.42 (21)	0.48
Professional athletics		4.64 (7)		20.18 (23)	15.63***
K-12 Education		11.92 (18)		1.75 (2)	9.62**
*Degree requirements*					
High school diploma		13.25 (20)		18.58 (21)	9.73*
Associate’s degree		3.31 (5)		4.42 (5)	
Bachelor’s degree		25.17 (38)		38.05 (43)	
Advanced degree		58.28 (88)		38.94 (44)	

Notes:

1 Scales are average score on all non-missing items;

2 Career aspirations are not mutually exclusive and therefore percentages do not add to 100;

* = *p* < 0.05;

** = *p* < 0.01;

*** = *p* < 0.001; Two-tailed tests.

**Table 3:** Logistic regression of career aspirations on independent variables, TRY-IT! (*n* = 265).

	Health and Medicine		Science and Technology		Creative and Performing Arts	

	OR (CI)	OR (CI)	OR (CI)	OR (CI)	OR (CI)	OR (CI)
Female	3.34 (1.87–5.95)***	3.02 (1.64–5.57)***	0.21 (0.12–0.38)***	0.23 (0.12–0.44)***	2.15 (1.11–4.16)*	2.67 (1.30–5.49)**
Black	0.47 (0.25–0.87)*	0.51 (0.26–0.98)*	0.73 (0.38–1.41)	0.83 (0.40–1.71)	1.57 (0.82–3.00)	1.17 (0.58–2.36)
Free or reduced lunch	0.89 (0.46–1.71)	1.12 (0.55–2.24)	0.49 (0.25–0.97)*	0.63 (0.29–1.35)	0.89 (0.43–1.87)	0.76 (0.34–1.70)
Parents’ educ (yrs)	0.96 (0.90–1.02)	0.94 (0.88–1.00)	1.00 (0.93–1.07)	1.01 (0.93–1.09)	0.98 (0.91–1.05)	0.99 (0.92–1.07)
School self-esteem		1.43 (1.00–2.04)*		0.71 (0.49–1.03)		0.75 (0.52–1.05)
Science self-concept		0.79 (0.55–1.12)		2.16 (1.38–3.37)***		1.02 (0.70–1.48)
Confidence in courses		1.38 (0.96–1.96)		0.74 (0.50–1.10)		0.69 (0.47–1.00)*
Enrichment		1.07 (0.97–1.25)		1.29 (1.08–1.55)***		0.98 (0.82–1.17)
*LRX*^2^	24.06***	36.12***	39.39***	67.24***	8.32*	18.96*
Pseudo R^2^	0.07	0.11	0.12	0.22	0.03	0.07

	**Law and criminal justice**		**Professional athletics**		**K–12 Education**	

	OR (CI)	OR (CI)	OR (CI)	OR (CI)	OR (CI)	OR (CI)

Female	0.77 (0.40–1.47)	0.73 (0.37–1.46)	0.16 (0.06–0.39)***	0.14 (0.05–0.38)***	7.38 (1.67–32.6)***	8.88 (1.95–40.6)**
Black	1.22 (0.61–2.44)	1.34 (0.64–2.79)	3.01 (1.29–7.01)**	2.39 (0.97–5.85)	1.50 (0.56–4.04)	1.47 (0.50–4.25)
Free or reduced lunch	1.35 (0.61–2.98)	1.23 (0.53–2.85)	1.51 (0.58–3.95)	1.26 (0.41–3.84)	0.95 (0.30–2.97)	0.79 (0.24–2.62)
Parents’ educ (yrs)	1.00 (0.93–1.08)	0.99 (0.92–1.07)	1.01 (0.93–1.11)	1.04 (0.94–1.16)	1.01 (0.90–1.13)	1.02 (0.91–1.15)
School self-esteem		1.10 (0.74–1.63)		1.00 (0.62–1.62)		0.68 (0.40–1.13)
Science self-concept		1.00 (0.67–1.49)		0.55 (0.34–0.90)*		0.95 (0.55–1.66)
Confidence in courses		1.37 (0.91–2.06)		0.91 (0.57–1.46)		1.43 (0.79–2.56)
Enrichment		0.85 (0.71–1.02)		0.99 (0.78–1.26)		0.80 (0.61–1.06)
*LRX*^2^	1.74	7.15	24.49***	31.71***	12.04*	17.69*
Pseudo R^2^	0.01	0.03	0.13	0.17	0.08	0.13

Notes: Scales are X-standardized, confidence intervals in parentheses;

* = *p* < 0.05;

** = *p* < 0.01;

*** = *p* < 0.001.

**Table 4:** Linear regression of years of education required for career aspirations on independent variables, TRY-IT! (*n* = 265).

	b (s.e.)	b (s.e.)
Female	1.74 (0.48) ***	1.77 (0.48) ***
Black	−1.30 (0.52) **	−0.71 (0.53)
Free or reduced lunch	−0.70 (0.58)	−0.09 (0.58)
Parents’ educ (yrs)	−0.01 (0.06)	−0.05 (0.06)
School self-esteem		0.19 (0.27)
Science self-concept		0.50 (0.28)
Confidence in courses		0.68 (0.28) **
Enrichment		0.17 (0.13)
Constant	17.28	15.99
*F*	5.37 ***	6.26 ***
*R*^2^	0.08	0.18

Note:

* = *p* < 0.05;

** = *p* < 0.01;

*** = *p* < 0.001.
